# Life-Threatening Necrotizing Pneumonia with Panton–Valentine Leukocidin-Producing, Methicillin-Sensitive *Staphylococcus aureus* in a Healthy Male Co-Infected with Influenza B

**DOI:** 10.3390/idr14010002

**Published:** 2021-12-26

**Authors:** Sara Agnete Hjort Larsen, Kasper Kyhl, Sharmin Baig, Andreas Petersen, Marita Reginsdóttir av Steinum, Sissal Clemmensen, Elin Jensen, Torkil á Steig, Shahin Gaini

**Affiliations:** 1 Medical Department, National Hospital Faroe Islands, 100 Tórshavn, Faroe Islands; kasperkyhl@gmail.com (K.K.); marsn@ls.fo (M.R.a.S.); lstorst@ls.fo (T.á.S.); shaga@ls.fo (S.G.); 2 Department of Cardiology, Copenhagen University Hospital, 2100 Rigshospitalet, Denmark; 3 Bacteria, Parasites & Fungi, Statens Serum Institute, 2300 Copenhagen, Denmark; BASJ@SSI.dk (S.B.); AAP@ssi.dk (A.P.); 4 Department of Radiology, National Hospital Faroe Islands, 100 Tórshavn, Faroe Islands; siscl@ls.fo; 5 Department of Intensive Care and Anesthesiology, National Hospital Faroe Islands, 100 Tórshavn, Faroe Islands; Elije@ls.fo; 6 Infectious Diseases Research Unit, Odense University Hospital, University of Southern Denmark, 5000 Odense, Denmark; 7 Centre of Health Research, Department of Science and Technology, University of the Faroe Islands, 100 Tórshavn, Faroe Islands

**Keywords:** Panton–Valentine leukocidin (PVL)-producing *Staphylococcus aureus*, necrotizing pneumonia, pneumonia, influenza B, PVL-producing methicillin sensitive *S. aureus*, co-infection, antimicrobial resistance

## Abstract

A previously healthy male was rushed into a hospital critically ill with confusion, sepsis, and acute respiratory distress syndrome only 43 h after having a normal chest X-ray and with blood samples showing only minimally elevated C-reactive protein. Two days earlier, the patient had returned to his home country, the Faroe Islands, from a 10-day work trip aboard a Scandinavian ship in Colombia. The diagnosis turned out to be an influenza B infection and necrotizing pneumonia with Panton–Valentine leukocidin (PVL)-producing methicillin-sensitive *Staphylococcus aureus* (MSSA). It was influenza season in Colombia but not in the Faroe Islands. The frequency of MSSA with PVL-encoding genes among pediatric infection patients is very low in the Kingdom of Denmark and Faroe Islands and very high in Colombia, and the frequency generally varies highly by region. The patient in this case now suffers severe sequelae from the infection. With this case, we would like to remind clinicians of this rare but severe condition. PVL-producing *S. aureus* pneumonia should be considered in critically ill, previously healthy patients, especially during influenza season and if the patient has been traveling in countries with high frequencies of PVL-producing *S. aureus.*

## 1. Introduction

PVL-positive *Staphylococcus Aureus (S. aureus)* can cause rapidly progressing necrotizing pneumonia even in young and previously healthy people. PVL-positive *S. aureus* pneumonia is often preceded by influenza infection. Here, we present the case of a previously healthy 47-year-old male who was diagnosed with PVL-positive methicillin-sensitive *S. aureus* (MSSA) necrotizing pneumonia preceded by influenza B infection after he returned to the Faroe Islands from a 10-day work trip aboard a Scandinavian ship in Colombia. He had a normal chest X-ray (CXR) and normal blood samples except for minor C-reactive protein (CRP) elevation (11 mg/L) only 43 h before he was admitted directly to the intensive care unit (ICU) with septic shock and acute respiratory distress syndrome (ARDS).

## 2. Case Presentation

A 47-year-old Faroese male without pre-existing comorbidities visited his general practitioner due to a worsening of influenza-like symptoms. A couple of hours earlier, he had arrived in the Faroe Islands by plane from a 10-day maintenance work period aboard a Scandinavian ship in Colombia. During the last 5 days of his stay in Colombia, he had been feeling ill with fever, chills, and coughing. Three days before his return to the Faroe Islands, he had gone to a general medicine consultation in Colombia. A physical examination revealed a body temperature of 37 °C, blood pressure of 90/60 mm Hg, heart rate of 74 beats/min, and oxygen saturation of 96%. The standard blood test results were normal. The patient received intravenous fluids with dipyrone analgesic before he was discharged, with the recommendation to see a doctor again as soon as he returned to the Faroe Islands.

**Figure 1:**
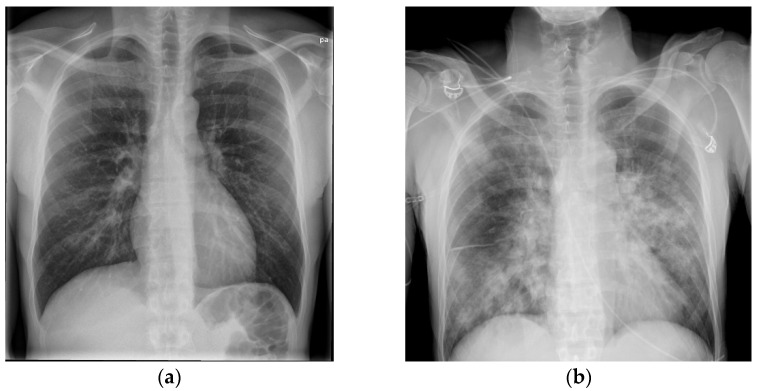
(**a**,**b**) The rapid onset of the severe disease is illustrated here. (**a**) shows a normal CXR from day 1, 2 days prior to admission. (**b**) shows a CXR with diffuse consolidation on the day of admission (day 3).

The general practitioner in the Faroe Islands sent him to the radiology department as an out-patient to get a CXR. During the visit to the radiology department, he began to feel lightheaded and nearly fainted. The staff sent him to the emergency department (ED). In the ED, he mentioned that he had felt dyspnoeic and had had productive coughing for 2 days, but that he was feeling better, and the dizziness probably was linked to lack of sleep and exhaustion from the long journey from Colombia to the Faroe Islands. A physical examination revealed a body temperature of 38.9 °C, blood pressure of 134/84 mm Hg, heart rate of 67 beats/min, and oxygen saturation of 98%. The CXR was normal (Figure 1a), and his blood tests showed hemoglobin levels of 7.7 mmol/L, leukocyte levels of 5 × 10^9^/L, and CRP levels of 11 mg/L (Table 1). The patient was a non-smoker and had no history of excessive alcohol consumption. He lived with his wife and children and was a marathon runner in his spare time. The patient was discharged from the ED and advised to contact a doctor if the symptoms worsened or did not get better within a couple of days.

In the morning 2 days later, he was rushed to the hospital by ambulance. He had woken up with labored breathing and episodes of bloody diarrhea and was confused and unable to recognize his own children. Physical examination at admission revealed a critically ill and confused patient with rattling respiration and a respiratory frequency of 50 breaths per minute. He was sweating and appeared dehydrated. The skin was without petechiae or ecchymosis. He presented a body temperature of 38.7 °C, heart rate of 120 beats per minute, oxygen saturation of 93%, and blood pressure of 84/56 mm Hg.

An arterial blood gas test showed a normal pH of 7.40, PaCO_2_ level of 5.0 kPa, decreased PaO_2_ level of 9.5 kPa, and elevated lactate level of 3.0 mmol/L. Blood tests showed a hemoglobin level of 8.7 mmol/l, leucocytes level of 1.1 × 10^9^/L, thrombocyte level of 113 × 10^9^/L, creatinine level of 160 µmol/L, and CRP level of 370 mg/L (Table 1). The CXR now showed diffuse bilateral consolidation (Figure 1b). The diagnoses up front were severe community-acquired pneumonia (CAP), septic shock, and ARDS. Disseminated intravascular coagulation (DIC) in progress was first suspected with the symptom of bloody diarrhea in mind, but the blood test results did not suggest severe DIC. The patient was transferred directly to the ICU to get intubated and receive fluid resuscitation, inotrope support, and antibiotic therapy (Table 2).

Microbiological tests collected on admission revealed *S. aureus* in the blood and sputum cultures. Polymerase chain reaction (PCR) testing of the sputum was positive for influenza B and negative for atypical pulmonary bacterial pathogens. Blood tests were furthermore negative for HIV and malaria. The urine culture was negative. Urine antigen detection for *Legionella pneumophila* and *Streptococcus pneumoniae* was negative. Resistance testing of the *S. aureus* isolate concluded it to be methicillin-sensitive, and molecular testing of the isolate showed the *S. aureus* to be PVL-positive (analyzed at the Danish National Reference Laboratory for Clinical Microbiology, Statens Serum Institut, Copenhagen, Denmark).

**Figure 2:**
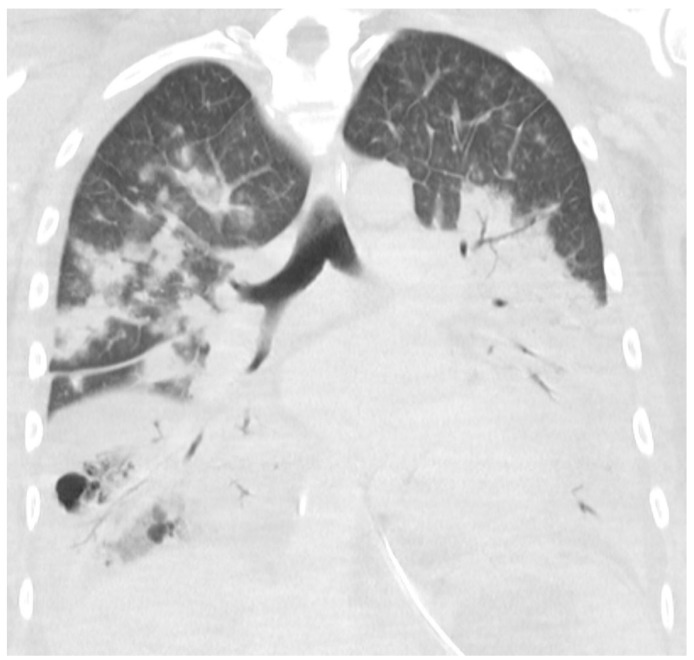
CT scan of the chest with coronal reconstruction. Massive bilateral consolidation with air bronchogram and minimal pleural effusion at day 4.

Transthoracic echocardiography showed a left ventricular ejection fraction of 40%. No other abnormalities and no signs of endocarditis were found. A CT scan of the cerebrum, neck, chest, abdomen, and pelvis showed massive bilateral lung infiltrates, atelectasis, and ARDS changes with small amounts of pleural fluids and ascites. No abscesses or infectious foci outside the lungs were found. Figure 2 shows a CT scan of the chest 4 days after admission.

Two and a half weeks after admission, the patient was still critically ill and intubated with a great need for an oxygen supply. He developed acute tubulointerstitial nephritis alongside existing ARDS and was transferred to tertiary care at Copenhagen University Hospital, Rigshospitalet in Denmark for acute hemodialysis and further intensive care treatment. He was hospitalized in Denmark for 5 weeks with a decreasing need for dialyses and ventilation and then transferred back to the Faroe Islands, where he was hospitalized for a further 6 weeks.

**Figure 3:**
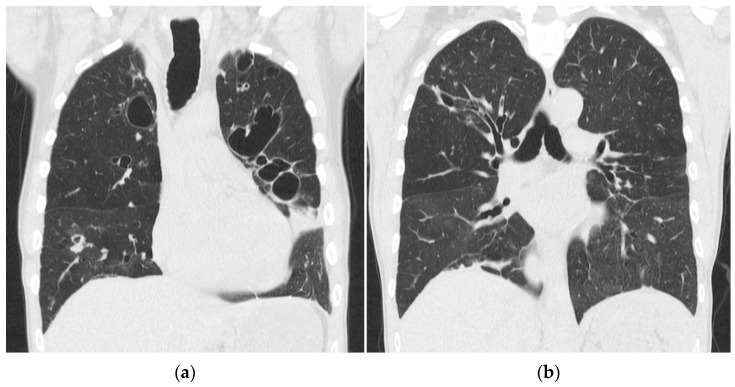
(**a**,**b**) CT scan of the chest with coronal reconstruction, showing bilateral pulmonal cysts, bronchiectasis, consolidation and dilation of the trachea, 5 months after admission.

After 13 weeks of hospitalization in total, he was discharged with home oxygen therapy and offered rehabilitation. Five months later, he did not need home oxygen therapy but was still limited in his daily life because of troubled and wheezing breathing. A spirometry showed severely reduced lung capacity with a forced vital capacity (FVC) of 2.56 L (51.5% of the expected capacity) and signs of emphysema and obstruction. A CT scan of the chest showed bilateral pulmonal cysts, bronchiectasis, consolidation, and dilation of the trachea (Figure 3). Scanning in the expiratory phase showed an almost complete collapse of the trachea and comprehensive air trapping, and he was diagnosed with tracheomalacia. Now, 4 years later, the patient is still followed in the Pulmonology Out-Patient Clinic at the National Hospital of the Faroe Islands. One and a half years after discharge, he was examined at a German thorax clinic to find out if he could benefit from a tracheal stent implantation, but an indication was not found. He now receives intermittent continuous positive airway therapy (CPAP), regular pneumococci, influenza vaccinations, and prophylactic antibiotics.

## 3. Materials and Methods

### Characterization of the Bacterial Isolate

The Department of Clinical Microbiology at the National Hospital of the Faroe Islands analyzed blood cultures for pathogens using the BD BATEC FX blood culture system, and microbial identification was performed using Bruker MALDI-TOF before resistance testing according to EUCAST guidelines. The following more specialized analyses were performed at the Danish National Reference Laboratory for Clinical Microbiology at the Statens Serum Institut in Copenhagen, Denmark. The isolate was spa-typed using a previously described protocol [1]. Genomic DNA was purified with MagNA Pure 96 DNA and a Viral Small Volume Kit (Roche Diagnostics A/S).

Short-read whole-genome sequencing was performed using a 300-cycle kit on Illumina’s NextSeq platform using a previously described pipeline [2]. Resistance and virulence genes were found using ResFinder and VirulenceFinder, respectively [3,4]. Single-nucleotide polymorphisms (SNPs) were identified with NASP version v.1.2.0, and core genome MLST (cgMLST) was assigned using SeqSphere + v5.1.0 (Ridom GmbH, Münster, Germany; http://www.ridom.de/seqsphere/) (24 December 2021) [5,6]. The phylogenetic relationship to an international collection of *S*. *aureus* ST152 was determined [7].

## 4. Results

The bacterial isolate was typed to *spa* type t16641, MLST type ST152, and cgMLST type CT15644. Whole genome sequencing demonstrated the presence of the *blaZ* gene, but no other resistance markers, and the lukF/S-PV gene, encoding forPVL. The isolate grouped among other PVL-positive MSSA in a previously published phylogeny of 149 clonal complex 152 (CC152) *S*. *aureus* ST152 but with no close relationship to any other strain in the phylogeny (data not shown) [7].

## 5. Discussion

Necrotizing pneumonia is associated with PVL-producing *S. aureus* [8]. Necrotizing pneumonia is a rapidly progressive disease with a high lethality rate. It mainly affects healthy children or young adults, who initially present with influenza-like symptoms [8].

PVL toxin is a pore-forming *S. aureus* toxin which can induce the lysis of leukocytes, particularly neutrophils [9]. Its role in severe necrotizing diseases has been and still is being debated, since laboratory work which mainly used murine disease models reported very contrasting results. In 2010, Löffler et al. demonstrated that the toxin induces rapid activation and premature cell death in human and rabbit neutrophils but not in murine or simian cells. They demonstrated that PVL acts differently in cells of various species and has an important cytotoxic role in human neutrophils [9].

In 1999, Lina et al. noticed that patients with PVL-positive necrotizing pneumonia often had a predisposing viral infection [10]. Löffler et al. presented a theory on the role of influenza in PVL-positive necrotizing pneumonia [11]. When microorganisms such as influenza or *S. aureus* invade lung tissue, neutrophil granulocytes are the first cells recruited to the site of inflammation. They contain proteases and other compounds that are intended to degrade engulfed microorganisms, but when the neutrophile granulocytes are met by PVL toxin, rapid cell destruction is initiated, and the potent antimicrobial compounds in the neutrophile granulocytes are left over and can cause necrosis of the lung tissue, potentially leading to necrotizing pneumonia [11].

In this report, we presented a previously healthy male with influenza B and PVL-positive *S. aureus*. The patient was admitted to a hospital in the Faroe Islands in the northern hemisphere in early June, outside of the local influenza season, and influenza was therefore not a straightforward diagnosis [12]. Two days prior to admission to the ICU, he had arrived by plane from Colombia, where he had been for 10 days working on a ship. According to the WHO, the influenza activity in tropical southern America in 2017 was year-round, with one peak in January and one in early June. Influenza A (H3N2) predominated, but influenza B also circulated in the region, notably in Ecuador and Colombia [12]. The patient did go onshore as a tourist with his colleagues, and it is therefore highly likely that he was infected with influenza B in Colombia. On top of influenza, he got an infection with PVL-positive MSSA. Only 2 cases of PVL-positive MSSA have been reported on the Faroe Islands previously and 36 cases with PVL-positive MRSA.

According to Zanger et al., PVL is common in *S. aureus* imported from the tropics and subtropics [13]. In 2015, Correa-Jiménez et al. evaluated the frequency of PVL-encoding genes in MSSA from isolates from pediatric infection patients in a hospital in Cartagena, Colombia, a 4-hour drive from the harbor, where the Scandinavian ship had docked. They reported a 68% frequency of PVL genes in MSSA isolates, one of the highest in the world, exceeded only by the 81.1% rate reported in Mozambique [14]. Correa-Jiménez et al. mentions that the prevalence of PVL-encoding genes in MSSA varies by region, and in contrast to a high prevalence found in Colombia, Argentina, and African countries, low prevalence has been found in countries such as Spain and Portugal [14]. In a Danish study by Nielsen et al. from 2020, the percentage of PVL-encoding genes in pediatric patients with MSSA bacteremia was 1.9%, much lower than the frequency found in Colombia [15]. The numbers from Colombia and Denmark are not directly comparable, but the findings are consistent with the prevalence of PVL-encoding genes in MSSA being extremely varied around the world.

The patient presented in this case now suffers from severe sequelae after the necrotizing pneumonia and the long treatment with mechanical ventilation. With this case, we would like to remind clinicians to be aware of this rare but severe diagnosis. It is important to start the right treatment immediately, since necrotizing pneumonia is toxin-mediated, and as soon as toxins are released, they cannot be confined by antimicrobial treatments [11]. Necrotizing pneumonia should be considered in younger patients with pneumonia and bacteremia with rapidly progressing symptoms. We should especially be aware in the influenza season and if people have been traveling in places during active influenza season and with a high prevalence of PVL-positive *S. aureus*.

## 6. Conclusions

- This case illustrates the rapid progression of necrotizing pneumonia causing severe lung damage.;
- Necrotizing pneumonia should be considered in critically ill, previously healthy patients with rapidly progressing symptoms;
- Necrotizing pneumonia should be considered if the patient has been traveling in places with a high prevalence of PVL-positive *S. aureus* during influenza season.

## Figures and Tables

**Table 1:** Blood samples.

	Unit	Ref	Day 1	Day 3 ^1^	Day 4
**B-hemoglobin**	micromol/L	8.0–10.5	7.7	8.7	8.2
**B-leukocytes**	×10^9^/L	3.0–10.0	5	1.1	1.3
**B-thrombocytes**	×10^9^/L	120–350	145	113	46
**B-neutrophils**	×10^9^/L	2.0–8.0	3.08	0.45	0.28
**P-potassium**	mmol/L	3.5–4.6	3.4	3.3	4.7
**P-sodium**	mmol/L	137–144	133	127	142
**P-creatinine**	μmol/L	60–105	83	160	206
**P-carbamide**	mmol/L	3.2–8.1	-	14.4	17.4
**P-albumin**	g/L	35–52	-	26	24
**INR**		0.8–1.2	-	1.6	1.8
**APTT**	s	23–35	-	41	36
**CRP**	mg/L	<6	11	370	494

^1^ Day 3 is the day of admission. INR = international normalized ratio; APTT = activated partial tromboplastin time; CRP = C-reactive protein.

**Table 2:** Antibiotic therapy within the first 15 days. Day 3 is the day of admission. All antibiotics were administered intravenously.

**Day 3**	Benzylpenicillin 1.2 g × 4	Ciprofloxacin 400 mg × 2	-
**Day 3**	Meropenem 2 g × 3	Ciprofloxacin 400 mg × 2	-
**Day 4**	Meropenem 2 g × 3	Moxifloxacin 400 mg × 1 ^1^	-
**Day 6**	Meropenem 2 g × 3	Moxifloxacin 400 mg × 1	Clindamycin 600 mg × 3 ^2^
**Day 7**	Meropenem 2 g × 3	Moxifloxacin 400 mg × 1	Clindamycin 600 mg × 3
**Day 8**	Meropenem 2 g × 3	Moxifloxacin 400 mg × 1	Clindamycin 600 mg × 3
**Day 11**	Meropenem 2 g × 3	Moxifloxacin 400 mg × 1	Clindamycin 600 mg × 3
**Day 12**	Meropenem 2 g × 3	-	Clindamycin 600 mg × 3
**Day 15**	-	-	Clindamycin 900 mg × 3 ^3^

^1^ Moxifloxacin was added on day 4 to include coverage of *Chlamydophila pneumonia.* ^2^ Clindamycin was added on day 6 as the patient fulfilled the criteria for toxic shock syndrome but without skin affection. ^3^ The clindamycin dose is increased to 900 mg × 3.

## Data Availability

No appliable.
